# Functional and structural changes in the visual pathway in multiple sclerosis

**DOI:** 10.1002/brb3.1467

**Published:** 2019-11-16

**Authors:** María Carcelén‐Gadea, Carlos Quintanilla‐Bordás, Alicia Gracia‐García, Carolina García‐Villanueva, Nicolás Jannone‐Pedro, Lourdes Álvarez‐Sánchez, Laura Vilaplana‐Domínguez, Trinidad Blanco‐Hernández, José Miguel Pons‐Amate, Angeles Cervelló‐Donderis

**Affiliations:** ^1^ Department of Neurology Consorcio Hospital General Universitario de Valencia Valencia Spain; ^2^ Department of Ophthalmology Consorcio Hospital General Universitario de Valencia Valencia Spain; ^3^ Department of Clinical Neurophysiology Consorcio Hospital General Universitario de Valencia Valencia Spain

**Keywords:** follow‐up, multiple sclerosis, optic neuritis, optical coherence tomography, transorbital sonography, visual evoked potentials

## Abstract

**Introduction:**

Multiple sclerosis (MS) is a heterogeneous disease with an unpredictable course. Visual pathway is a target of the disease and may reflect mechanisms that lead to disability. Structural and functional changes in the visual pathway may be studied by noninvasive techniques such as optical coherence tomography (OCT), visual evoked potentials (VEP), or B‐mode transorbital sonography (TOS).

**Objectives:**

The aim is to assess changes in the visual pathway in eyes of MS patients with and without a history of optic neuritis over a 3‐year period and to explore their relationship with disability.

**Materials and Methods:**

In total, 112 eyes from 56 patients with relapsing MS were recruited: 29 with, and 83 without a history of ON (hON and nhON, respectively). Several parameters were measured by OCT, VEP, and TOS. Baseline measurements were also compared to 29 healthy controls. At 36 months, measurements were repeated in all eyes.

**Results:**

At baseline, all tests showed significant differences in optic nerve structure and function in both patient cohorts in all the parameters studied, suggestive of more impairment of the visual pathway among the hON cohort. OCT showed significant differences between healthy controls and the nhON cohort. At 36 months, the nhON cohort showed significant changes by OCT, VEP, and TOS suggestive of further visual pathway impairment. OCT measurements also correlated with baseline EDSS among the nhON cohort.

**Conclusions:**

OCT is the most suitable technique and outperforms VEP and TOS to detect subclinical damage in the visual pathway. It discriminated MS patients from healthy controls and showed a progressive decline in optic nerve thickness over time among these patients.

## Graphical abstract

We assesed the visual pathway in MS patients for 3 years using optical coherence tomography (OCT), visual evoked potentials and transorbital sonography. All techniques showed impairment of visual pathway over time and OCT resulted the best to detect subclinical damage.

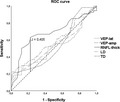

## 1 INTRODUCTION

Multiple sclerosis (MS) is a heterogeneous disease with a variable clinical course. Much research has been focused on biomarkers that could predict patients' clinical course and long‐term disability. Study of the visual pathway has been a field of interest as it may reflect brain changes that contribute to patient disability (Costello, 2013).

The retina and the optic nerve are functionally eloquent structures, known to be important targets of the disease even in patients without optic neuritis (ON). Their study may portray brain axonal damage, with the advantage of being easily accessible by inexpensive and noninvasive techniques such as optical coherence tomography (OCT), visual evoked potentials (VEP), and transorbital sonography (TOS) of the retrobulbar optic nerve. The aim of the study was to evaluate the suitability of these three techniques to monitor visual pathway changes over a 3‐year period in MS patients and to explore their relationship with disability.

## 2 PATIENTS AND METHODS

This was a prospective, 36‐month observational study of a total of 112 eyes from 56 patients with MS, of which 29 had history and 27 did not have a history of ON. Patients were recruited from the MS clinic in the Neurology Service at Hospital General Universitario de Valencia. Eyes of patients were segregated into two cohorts according to the history of optic neuritis (hON eye cohort, *n* = 29) or non‐history of ON (nhON eye cohort, *n* = 83).

All eyes underwent VEP, OCT, and TOS measurements at baseline and at 36 months. Correlation between these findings and clinical variables was explored. At the beginning of the study, baseline measurements were also compared to a healthy group.

To ensure comparability of the cohorts, baseline demographic and clinical variables of the patients were collected and compared before eye segregation. These included disease duration, accumulated number of relapses, baseline neurological disability as measured by the EDSS, baseline MRI T2 lesion and T1 Gd—enhancing lesion count, and the presence of disease‐modifying treatment (Table 1).

**Table 1:** Baseline clinical characteristics of the patients before grouping into eye groups

	Mean ± *SD* [range]		
	MS patients without history of ON (*n* = 27)	MS patients with history of ON (hON cohort, *n* = 29)	Healthy controls (*n* = 29)
Age	36.30 ± 7.24 [18–50]	36.20 ± 5.94 [22–47]	35 ± 5.84 [24–50]
Number of female patients (%)	18 (66.7%)	23 (79.3%)	20 (69.0%)
Disease duration (years)	5.48 ± 3.83 [1–14]	6.55 ± 4.28 [1–14]	
Cumulative number of relapses	4.07 ± 1.59 [2–8]	3.66 ± 1.68 [2–10]	
EDSS score	1.83 ± 0.76 [1–3.5]	2.00 ± 0.91 [1–4]	
MRI T2 lesion count	1.63 ± 1.69	1.13 ± 1.51	
MRI T1 Gd‐enhancing lesion count	0.25 ± 0.58	0.13 ± 0.575	
Presence of disease‐modifying treatment	25	26	

Abbreviations: hON, history of ON; MS, multiple sclerosis; nhON, no history of ON; ON, optic neuritis.

### 2.1 Patients

**Figure 1:**
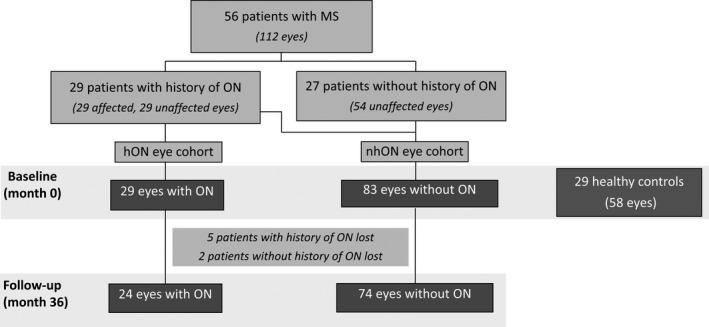
Outline of the study population

We included patients younger than 50 years with relapsing–remitting MS (RRMS) diagnosis according to the 2005 McDonald criteria (Polman et al., 2005) with a score on the Expanded Disability Status Scale (EDSS) of less than or equal to 4. Measurements were taken at least 6 months after last ON episode. Patients with causes of vision loss or retinal damage not attributable to MS were excluded (Figure 1).

Among the hON cohort, recordings were taken at least 6 months after the onset of ON symptoms. ON was diagnosed by the clinical criteria of ocular pain with eye movements and a decrease in visual acuity referred by the patient and confirmed in the ophthalmological examination. During the acute phase of ON, all the patients received 1 g of intravenous methylprednisolone for 3–5 days.

The study was completed ensuring compliance with current ethical and legal standard (Declaration of Helsinki) and approved by the local ethics and research committee. All the participants signed their informed consent prior to inclusion in the study.

### 2.2 Instrumental variables measured

A set of nine measurements were taken for each eye. These were latency (VEP‐lat) and amplitude (VEP‐amp) of the p100 wave of the VEP, global and quadrant‐based (inferior, nasal, superior, temporal) retinal nerve fiber layer thickness (RNFL‐thick, I‐RNFL‐thick, N‐RNFL‐thick, S‐RNFL‐thick, and T‐RNFL‐thick, respectively) measured by OCT, longitudinal diameter (LD), and transversal diameter (TD) of the retrobulbar optic nerve measured by TOS.

### 2.3 Technique

#### 2.3.1 Visual evoked potentials

VEPs were measured with a 10‐channel Synergy monitor using the pattern or checkerboard technique. All examinations were performed by an experienced neurophysiologist.

The 10–20 International System was used for electrode placing (American Clinical Neurophysiology Society, 2006), and electrodes were connected to a Dantec Keypoint 6ch EP amplifier (Natus Medical). The visual stimulus, featured on a 25 × 33 cm Sony Trinitron Multiscan monitor (model 17SE), was a checkerboard pattern, which reverses every 1.8 s. Mean luminance of the pattern was 50 cd/m^2^ and contrast >80%. All measurements were in accordance with the international guidelines and the International Society for Clinical Electrophysiology of Vision (Odom et al., (2016)). Three square sizes were used, 18 mm (60′), 9 mm (30″), and 4.5 mm (15′), respectively, each with a run of 200 stimulations to ensure the lowest possible noise level. Analysis time was maintained at 300 ms and the rejection level at 200 μV.

#### 2.3.2 Transorbital ultrasound

Transorbital ultrasound (TOS) measurements of the retrobulbar optic nerve were taken with a color duplex ultrasound with a 10 Hz linear probe (Esaote MyLab™ 25 Gold). To measure the LD, the transducer was placed on the upper eyelid and the measurement was taken 3 mm from the papilla; to measure the TD, the probe was placed on the lower eyelid, perpendicular to the facial plane.

#### 2.3.3 OCT

RNFL‐thick was measured by a spectral domain OCT (SD 3D OCT 2000 Top Con) with a 3D acquisition protocol (6.0 × 6.0 mm–512 × 128). Patients were scanned under photopic conditions with ceiling lights and without pupil dilation. An experienced grader masked for clinical presentation checked all images for sufficient image quality in line with the OSCAR‐IB criteria (Schippling et al., 2015) and manually corrected segmentation in case of errors. All scans were carefully checked by an ophthalmologist.

### 2.4 Statistics

Patients who met the inclusion criteria were consecutively recruited. Statistical analysis was carried out using the Statistical Package for the Social Sciences (SPSS) version 23. In the univariate descriptive analysis, quantitative variables are expressed as the mean ± the standard deviation (*SD*) and the qualitative variables were expressed as percentages. The Student's *t* test or the equivalent nonparametric test (Mann–Whitney *U* test) when normality of the variable could not be assumed, were used to check the homogeneity of the cohorts and to analyze the differences between their two means. Differences in proportions were evaluated with the *χ* ^2^ test or the Fisher's exact test, as appropriate. The results at baseline and at 36 months were compared using the two‐tailed Student's *t* test for paired normally distributed data and Wilcoxon test, otherwise.

The association between the different variables was evaluated with the Pearson correlation coefficient or Spearman coefficient according to the normality of the two variables in the corresponding subgroup.

## 3 RESULTS

### 3.1 Baseline

The baseline clinical and demographic characteristics of the three groups are summarized in Table 1. All the groups were homogeneous in terms of their demographic variables (sex and age) and medical history (alcohol and tobacco consumption, hypertension, diabetes mellitus, dyslipidemia, and other neurological or autoimmune diseases). Also, patients with and without a history of ON did not show significant differences in terms of clinical variables (disease duration, accumulated number of relapses, baseline EDSS, baseline MRI T2 lesion and T1 Gd—enhancing lesion count, and the presence of disease‐modifying treatment).

Baseline measurements (Table 2) revealed that hON eyes had statistically significant longer VEP‐lat (*p* = .0009) and smaller RNFL‐thick (*p* = .0012), but also a tendency for lower VEP‐amp (*p* = .0484), LD (*p* = .0242), and TD values (*p* = .0213) with respect to nhON eyes.

**Table 2:** Healthy controls, nhON, and hON cohort baseline and 36‐month follow‐up measurements

	Mean ± S.D.				
	Eyes from healthy controls (*n* = 58)	nhON eyes cohort		hON eyes cohort	
		Baseline (*n* = 83)	Follow‐up (*n* = 74)	Baseline (*n* = 29)	Follow‐up (*n* = 24)
RNFL‐thick (µm)	104.28 ± 8.15	97.31 ± 13.10	89.50 ± 14.89	93.50 ± 11.89	81.50 ± 15.22
T‐RNFL (µm)	76.34 ± 10.65	70.20 ± 13.88	63.54 ± 13.32	61.42 ± 13.12	53.21 ± 12.97
S‐RNFL (µm)	125.79 ± 11.18	114.53 ± 16.92	109.20 ± 13.70	110.63 ± 21	101.67 ± 16.31
I‐RNFL (µm)	132.017 ± 15.94	121.72 ± 20.20	114.64 ± 20.12	116.04 ± 16.09	102.04 ± 21.00
N‐RNFL (µm)	82.55 ± 14.00	84.32 ± 17.56	76.18 ± 16.86	82.79 ± 15.62	71.00 ± 17.64
TD (mm)	3.71 ± 0.28	3.48 ± 0.53	2.81 ± 0.49	3.26 ± 0.61	2.76 ± 0.62
LD (mm)	4.90 ± 0.26	4.08 ± 0.59	3.66 ± 0.35	3.88 ± 0.77	3.57 ± 0.18
VEP‐lat (ms)	103.89 ± 5.39	107.19 ± 9.93	108.98 ± 10.77	112.74 ± 15.09	108.66 ± 13.78
VEP‐amp (µV)	9.23 ± 3.15	8.26 ± 3.38	7.67 ± 2.93	8.44 ± 3.66	7.91 ± 3.22

Abbreviations: hON, history of ON; I‐RNFL, inferior quadrant retinal nerve fiber layer thickness; LD, longitudinal diameter of the optic nerve; nhON, no history of ON; N‐RNFL, nasal quadrant retinal nerve fiber layer thickness; RNFL‐thick, average retinal nerve fiber layer thickness; S‐RNFL, superior quadrant retinal nerve fiber layer thickness; TD, transverse diameter of the optic nerve; T‐RNFL, temporal quadrant retinal nerve fiber layer thickness; VEP‐amp, visual evoked potentials amplitude; VEP‐lat, visual evoked potentials latency.

A similar pattern was found when comparing nhON cohort to healthy controls, although only the decrease in RNFL‐thick was found significant (*p* = .0036). In addition, baseline EDSS was the only clinical variable that correlated negatively with baseline RNFL‐thick in the nhON cohort (*r* = −.4161, *p* = .02).

### 3.2 Diagnostic performance of OCT, VEP, and TOS

**Figure 2:**
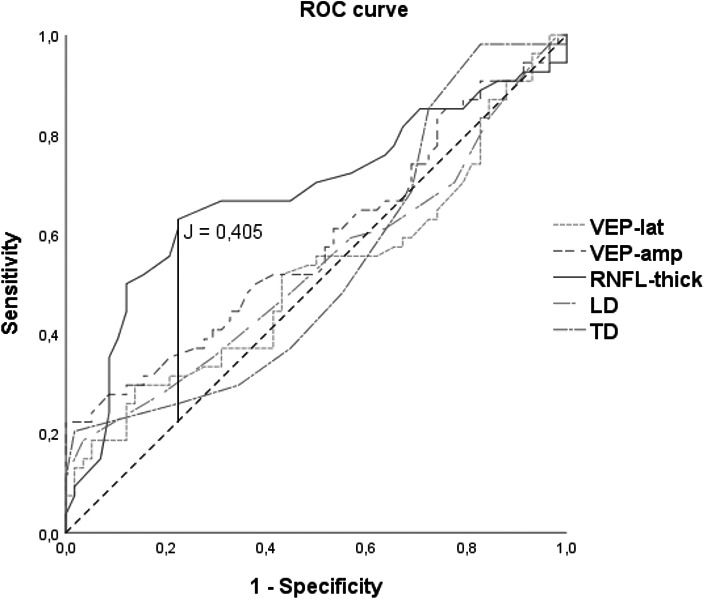
Comparison of ROC curves of the different tests in healthy controls and in patients with MS without ON. AUC values: VEP‐lat: 0.518; VEP‐amp: 0.583; RNFL‐thick: 0.681; LD: 0.532; TD: 0.534. RFNL‐thick Youden's index (J) = 0.405, sensitivity = 0.630, 1‐specificity = 0.224 (marked with a vertical line) for a cutoff value of 99.50 μm. LD, longitudinal diameter of the optic nerve; RNFL‐thick, average retinal nerve fiber layer thickness; TD, transverse diameter of the optic nerve; VEP‐amp, visual evoked potentials amplitude; VEP‐lat, visual evoked potentials latency

To evaluate the ability of OCT, VEP, and TOS to discriminate between nhON from controls (and thus, the capability of detecting subclinical damage), we carried out a receiver operating characteristic (ROC) analysis for each test (Figure 1). The area under the curve (AUC) values were 0.681 (RNFL‐thick), 0.518 (VEP‐lat), 0.583 (VEP‐amp), 0.532 (LD), and 0.534 (TD). Furthermore, Youden's index was used to calculate the optimal cutoff point for the RNFL‐thick test, which was 0.405 (sensitivity = 0.630, specificity of 0.776) for a cutoff value of 99.50 μm (Figure 2).

### 3.3 Comparison of baseline and 36‐month follow‐up measurements

During follow‐up, five patients with a history of ON and two patients without a history of ON were lost, resulting in a loss of five eyes in the hON and nine in the hON cohort. No patient presented an episode of ON between the two examinations. After follow‐up, the groups remained homogeneous in terms of demographic and clinical variables. Final EDSS was 2.12 ± 1.25 and 1.98 ± 0.92 (*p* = .364), and the number of relapses was 4.08 ± 2.06 and 4.44 ± 1.75 (*p* = .493) in the group of patients without history and with a history of ON, respectively.

Values of repeated follow‐up measurements are represented in Table 2. Statistical significance of follow‐up values with respect to baseline is shown in Table 3.

1. In the nhON cohort, there was a statistically significant elongation in the VEP‐lat and a statistically significant decrease in the VEP‐amp, RFNL thickness (both globally and by quadrants), LD, and TD (VEP‐lat, *p* = .027; VEP‐amp, *p* = .021; RNFL‐thick, *p* < .001; T‐RNFL‐thick, *p* < .001; S‐RNFL‐thick, *p* < .001; I‐RNFL‐thick, *p* = .04; N‐RNFL, *p* < .001; LD, *p* < .001; TD, *p* < .001).
2. In the hON cohort, there was a statistically significant decrease in RNFL thickness (both globally and by quadrants), LD, and TD (RNFL‐thick, *p* = <.001; T‐RNFL‐thick, *p* = <.001; S‐RNFL‐thick, *p* = .03; I‐RNFL‐thick, *p* = .01; N‐RNFL‐thick, *p* = <.001; LD, *p = *.028; TD, *p* = .002).

**Table 3:** Statistical significance when comparing the data at baseline and at 3‐year follow‐up

	*p*‐Value								
	VEP‐lat	VEP‐amp	LD	TD	RFNL‐thick	T‐RFNL‐thick	S‐RFNL‐thick	I‐RFNL‐thick	N‐RFNL‐thick
Eyes of nhON cohort (*n* = 74)	.027	.021	<.001	<.001	<.001	<.001	<.001	.04	<.001
Eyes of hON cohort (*n* = 24)	ns.	ns.	.028	.002	<.001	<.001	.03	.01	<.001

Abbreviations: hON, history of ON; I‐RNFL, inferior quadrant retinal nerve fiber layer thickness; LD, longitudinal diameter of the optic nerve; nhON, no history of ON; N‐RNFL, nasal quadrant retinal nerve fiber layer thickness; ns, non‐significant; RNFL‐thick, average retinal nerve fiber layer thickness; S‐RNFL, superior quadrant retinal nerve fiber layer thickness; TD, transverse diameter of the optic nerve; T‐RNFL, temporal quadrant retinal nerve fiber layer thickness; VEP‐amp, visual evoked potentials amplitude; VEP‐lat, visual evoked potentials latency.

## 4 DISCUSSION

In our series, VEP, OCT, and TOS discriminated MS patients with ON from those without it, suggesting that these techniques are sensitive enough to detect structural and/or functional changes produced in the optic nerve after an acute ON episode. However, of the three techniques, OCT yielded the best performance and was the only that could discriminate between MS eyes without a history of ON (nhON group) and healthy controls, as depicted by the ROC curve (Figure 1). With this test, a fall in RNFL‐thick below the cutoff value of 99.50 µm yielded a sensitivity of 0.630 and a specificity of 0.776 to detect subclinical damage. Also, after 3 years, thinning of the optic nerve could be detected by OCT and TOS in eyes despite no acute episodes having occurred during this interval. This is in line with previous prospective studies that confirm that MS patients experience loss of retinal ganglion cells and therefore thinning of the RNFL due to axonal loss in the optic nerve, even in the absence of ON (Garcia‐Martin et al., 2017, 2011; Petzold et al., 2010). Several hypotheses have been proposed to explain this phenomenon, including trans‐synaptic retrograde degeneration caused by lesions affecting the posterior visual pathways, primary damage to the retina, and subclinical demyelination in the optic nerve (Vidal‐Jordana, Sastre‐Garriga, & Montalban, 2012).

Thus, our work provides further the evidence that OCT measurements could act as a marker for global axonal damage in visually asymptomatic MS patients (Alonso, Gonzalez‐Moron, & Garcea, 2018). Moreover, this was reinforced by the fact that RNFL thickness measurement by OCT correlated with EDSS at baseline.

Our data also confirm recent studies that TOS demonstrates optic nerve atrophy regardless of the presence of prior ON (Candeliere Merlicco et al., 2018; Pérez Sánchez, 2018). However, this was the least sensitive of the three techniques to detect MS patients from healthy controls as demonstrated by the AUC values in the ROC curve (AUC of LD: 0.532; TD: 0.534).

VEP, however, could only detect worsening of optic nerve function (expressed by VEP‐lat elongation and VEP‐amp decline) in nhON eyes but not in hON eyes. A possible explanation is that the previous acute event could represent a floor effect of on the neurodegenerative process. In addition, neuroplasticity after an acute ON could result in some functional recovery, expressed by some improvement of the VEP amplitude and latency, although the underlying mechanisms are not well known (Klistorner et al., 2010, 2012).

Alteration of VEPs in patients without a history of ON may be useful as a supporting criterion in the diagnosis of MS because these changes may indicate subclinical damage in the visual pathway. Additionally, the functional deterioration we observed at the 3‐year follow‐up suggests that there is progressive functional damage to this pathway, even when visually asymptomatic. To corroborate these data and obtain more robust results, further studies with extended follow‐up periods and/or a larger sample size would be desirable. Also, the use of multifocal VEPs with higher sensitivity and specificity would be very useful in detecting these visual pathway abnormalities (Klistorner et al., 2008; Laron et al., 2009).

Given the data above, we conclude that OCT, VEP, and TOS may be valid to monitor visual pathway damage in MS patients and that OCT is the most sensitive and specific when compared to the other two. Considering the hypothesis that axonal loss is the predominant cause of disability in MS, it is predicted that RNFL thickness should be smaller in the progressive subtypes of the disease, which has already been shown by several authors (Alonso et al., 2018; Balk et al., 2016; Henderson et al., 2008; Pulicken et al., 2007; Ratchford et al., 2013). Interestingly, the rate of RNFL thinning has been found to be greater in active and earlier phases of the disease, which could reflect a plateau effect of advanced stages (Klistorner et al., 2008; Laron et al., 2009). Future studies intending to use any of these techniques as a measure of disability should be aware of eyes previously affected by an episode of ON, since baseline measurements could be already affected and thus, not reflect the overall axonal damage present. In our series, despite progressive damage to the optic nerve, no correlations were found between final EDSS and any of the parameters studied during follow‐up. Therefore, we cannot conclude that increased axonal damage corresponds to a higher disability. Similarly, other authors were also unable to demonstrate this association (Henderson et al., 2008; Oreja‐Guevara, Noval, Manzano, & Diez‐Tejedor, 2010), although recent studies established a relationship between RNFL thickness and risk of disability progression (Martínez‐Lapiscina et al., 2016), and rate of RNFL thinning and NEDA‐3 at 2 years (AUC = 0.8) (Pisa et al., 2017) There may be multiple reasons for this, including the short follow‐up period, patient heterogeneity, the small sample sizes used in most studies, or perhaps because EDSS is not sensitive enough to detect changes in disability when there is no motor impairment. Therefore, future studies should aim to use larger sample sizes, longer follow‐up times, and more sensitive scales capable of detecting subtler changes in disability.

## CONFLICT OF INTEREST

The authors have no conflict of interest to disclose.

## AUTHOR CONTRIBUTION

Carcelén‐Gadea M made substantial contributions to conception and design, or acquisition of data, or analysis and interpretation of data and was involved in drafting the manuscript or revising it critically for important intellectual content. Quintanilla‐Bordás C, Gracia‐García A, García‐Villanueva C, Jannone‐Pedro N, Alvarez‐Sánchez L, Vilaplana‐Domínguez L, Blanco‐Hernández T, Pons‐Amate JM, and Cervelló‐Donderis A contributed to acquisition of data and was involved in drafting the manuscript or revising it critically for important intellectual content.

## Data Availability

The data that support the findings of this study are available on request from the corresponding author. The data are not publicly available due to privacy or ethical restrictions.
